# Is generative artificial intelligence becoming addictive? An exploration among undergraduate students

**DOI:** 10.3389/fpsyg.2026.1887507

**Published:** 2026-07-28

**Authors:** Onur Isbulan, Ozkan Ergene, Eda Demirhan

**Affiliations:** 1 Department of Instructional Technologies, Sakarya University, Sakarya, Türkiye; 2 Department of Mathematics and Science Education, Sakarya University, Sakarya, Türkiye

**Keywords:** addiction, GAI dependency, generative artificial intelligence, scale development, undergraduate, validation

## Abstract

This study aimed to examine the multidimensional nature of Generative Artificial Intelligence (GAI) addiction among undergraduate students. For this purpose, firstly, the Generative Artificial Intelligence Addiction Scale (GAI-AS) was developed and administered to 919 undergraduate students. The GAI-AS was designed as a psychometrically valid instrument to assess addictive patterns of GAI use across four dimensions: salience, excessive dependency, mood modification, and functional impairment. The findings provide strong evidence of the scale's validity and reliability. Subsequently, the validated instrument was administered to 803 undergraduate students to investigate demographic and usage-related differences in GAI addiction tendencies. Findings revealed no significant differences in GAI-AS scores according to gender or type of GAI version used (free vs. paid). Grade-level differences were observed only in the dimension of excessive dependency, with freshman students reporting slightly lower scores than upper-class students. In contrast, frequency of GAI use emerged as a strong and consistent predictor, as daily users showed significantly higher scores across all four dimensions and on the total scale. Correlation analyses further demonstrated moderate positive associations among all GAI-AS sub-dimensions, supporting the conceptualization of GAI addiction as an interconnected behavioral and emotional phenomenon. These findings clarify GAI addiction as a psychological construct and provide a validated scale.

## 1 Introduction

In recent years, the acceleration of digitalization has positioned artificial intelligence (AI) systems as integral components of individuals' daily lives. According to recent reports by the (OECD 2024a,b, 2025), the adoption rate of AI technologies in businesses is increasing rapidly compared to previous years; they are now being utilized intensively, particularly in the information and communication, education, and healthcare sectors. This transformation is redefining not only economic productivity but also individuals' access to information, decision-making processes, and forms of social interaction. The ability of AI to provide human-like responses through speech-based interfaces (Troshani et al., 2021) and to interact continuously with users elevates it from being ordinary software to a perceived “interaction partner.” This situation has added a complex psychological dimension to the human-machine relationship, effectively focusing researchers' attention on “the behavioral effects of AI” (Montag et al., 2024).

Continuous interaction with AI systems can have significant implications for users' cognitive and emotional processes. Previous research on digital media has consistently shown that excessive technology use can lead to adverse outcomes, including stress, loneliness, distraction, and cognitive overload (Jung et al., 2019; Kuss and Griffiths, 2017). Similarly, the personalized and empathetic responses generated by chat-based AI systems may provide emotional support in the short term, yet they pose a risk of fostering social withdrawal and emotional detachment in the long term (Jose et al., 2025; Richet, 2025). De Freitas et al. (2026) explicitly demonstrated this paradox, showing that while “AI friends” reduced feelings of loneliness during short-term interactions, they reinforced withdrawal from real-world social relationships over time. Similarly, Herbener and Damholdt (2025) found that the frequency of chatbot use among adolescents was inversely related to social connectedness. Furthermore, Montag and Elhai (2025) suggested that positive attitudes toward AI may be associated with problematic usage patterns in certain individuals. Thus, the psychological effects of AI appear to be two-sided: it offers cognitive ease while simultaneously carrying a potential for emotional dependency (Montag and Ali, 2025; Brand et al., 2014).

Over the past decades, behavioral addiction research has provided a robust framework for understanding how engagement with digital technologies may transition from benign usage to dysfunctional or pathological patterns. The components model of addiction, outlined by Griffiths (2005) within a biopsychosocial framework, posits six core elements consisting of salience, mood modification, tolerance, withdrawal, conflict, and relapse that characterize addictive behaviors. While initially developed for gambling, this model has become a cornerstone in explaining various technological dependencies, including internet use, online gaming, and social networking (Kuss and Griffiths, 2017). Furthermore, early research on problematic internet use by scholars such as Caplan (2002) provides a strong foundation for applying this framework to emerging technology-based addictions, including the use of AI.

Against this theoretical backdrop, the specific affordances of generative AI (GAI) systems warrant a focused examination through the lens of behavioral addiction. Unlike earlier forms of digital engagement, which often rely on static content consumption or intermittent social feedback, GAI interactions are characterized by dynamic, personalized, and often anthropomorphic reciprocity. This unique capacity for “human-like” responsiveness may exacerbate specific addiction components, such as mood modification and salience, by satisfying users' social and emotional needs more potent than traditional internet applications. Consequently, adapting established frameworks, such as the components model, to the specific context of GAI is essential for conceptualizing and measuring the potential deregulated use of these emerging technologies.

### 1.1 GAI addiction: conceptualization and assessment

Within the current digital environment, generative and conversational GAI systems have introduced novel interactional affordances such as constant availability, personalized feedback, and anthropomorphic conversational styles, which may amplify addiction-risk features. For instance, chatbots and Large Language Model (LLM)-driven agents may fulfill social, emotional, and informational needs in a way that closely resembles human interaction (Epley et al., 2007). Empirical data now suggest that some users engage with GAI systems in ways analogous to the addictive use of other technologies. For example, a behavioral economics study of young adults in Bogotá demonstrated that using GAI tools showed a higher relative reinforcing value compared to monetary rewards under certain conditions (Robayo-Pinzon et al., 2025). Another study among Danish high school students found that those who used chatbots for friend-like conversations reported significantly higher loneliness and lower perceived social support compared to non-users (Herbener and Damholdt, 2025).

Additionally, new psychometric instruments are being developed to address this phenomenon (Akinwale et al., 2025; Chen et al., 2025; Maral et al., 2026; Morales-García et al., 2024; Zhang et al., 2025a). Morales-García et al. (2024) introduced the Dependence on Artificial Intelligence (DIA) scale, capturing dimensions related to emotional reliance and maladaptive AI use among undergraduate students. Likewise, Akinwale et al. (2025) developed the Artificial Intelligence Dependence Questionnaire (AIDQ) for undergraduate populations, with a primary focus on behavioral reliance. Zhang et al. (2025a) proposed the AI Chatbot Dependence Scale, which emphasized psychological attachment and habitual engagement with conversational agents. Huang et al. (2024), adapting items from the Smartphone Addiction Scale, introduced a brief five-item measure of AI dependence, however, this instrument involved limited scale development procedures and did not comprehensively operationalize addiction-related dimensions. Besides, the Conversational AI Dependence Scale (CAIDS), developed among Chinese undergraduate students, identified a four-factor structure: uncontrollability, withdrawal symptoms, mood modification, and negative impact and found significant associations with poorer psychological wellbeing (Chen et al., 2025). Meanwhile, the Problematic ChatGPT Use Scale (PCGUS) demonstrated positive correlations with internet gaming disorder and negative correlations with conscientiousness (Maral et al., 2026). These findings point to a key insight: while AI-mediated interaction may share mechanisms underlying social media or gaming addictions, such as reward expectancy, mood regulation, and escapism, it also introduces several distinct features.

*Anthropomorphic interaction*: When an GAI system simulates human-like conversation, the user may perceive it as a social partner, leading to increased emotional investment and a reduced sense of boundaries between the tool and companion (Epley et al., 2007).

*Always-on availability and instantaneous responses:* AI chatbots can provide immediate feedback and are accessible anytime, which reduces latency and may facilitate habitual usage (Troshani et al., 2021).

*Escapism and emotion-regulation function:* For users experiencing loneliness, social anxiety, or stress, AI chatbots may serve as an accessible emotional refuge, increasing the risk of dependency when used as a coping strategy rather than a tool (Yao et al., 2025).

*Functional impairment and loss of control:* The heavy use of GAI systems may lead to neglect of academic, occupational, or relational responsibilities, as well as difficulties in regulating time, indicating a shift from engaged use to problematic behavior (Richet, 2025).

Considering these factors, it is increasingly argued that “GAI addiction” should not simply be subsumed under existing technology-addiction categories but may warrant recognition as a distinct phenomenon with unique precursors, processes, and outcomes. Indeed, one review cautions that while claims of “ChatGPT addiction” abound, the evidence remains limited and fails to meet addiction criteria (Ciudad et al., 2025) consistently. However, the accelerated integration of conversational AI into daily life, along with early empirical signals of dependence, suggests the urgency of developing rigorous conceptualization and measurement instruments. This need aligns with Zhang et al.'s (2025b) call for the creation and validation of dedicated assessment instruments, particularly in light of the accelerating diffusion of conversational AI tools and the early indicators of GAI addiction documented in recent studies.

Existing scales related to digital addictions (e.g., Smartphone Addiction Scale, Social Media Addiction Scale) address general indicators of technology overuse; however, they do not adequately capture the emerging dimensions specific to interactions with GAI systems. Phenomena such as anthropomorphic engagement, co-creation with GAI tools, and the development of emotionally charged relationships with algorithmic agents extend beyond the conceptual scope of traditional measures of technology addiction. Although pioneering efforts, such as CAIDS and PCGUS, have begun to explore GAI-related dependency patterns, limitations concerning cultural adaptability and construct coverage remain evident. Recent instruments, including the DIA scale, the AIDQ, and CAIDS, have contributed valuable insights; yet, each tends to emphasize isolated aspects of GAI engagement, such as behavioral dependency or emotion regulation, or focuses on platform-specific use. Consequently, the literature continues to lack a theoretically integrative and psychometrically concise instrument capable of assessing GAI-related addiction within a comprehensive biopsychosocial framework. Addressing this identified gap necessitates the development of a measurement tool grounded explicitly in Griffiths' (2005) model, which encompasses the core dimensions of salience, excessive dependency, mood modification, and functional impairment within a unified structure.

Consequently, a valid, reliable, and culturally appropriate measurement tool encompassing behavioral, emotional, and cognitive dimensions is essential. Behavioral addiction research has conceptualized technology-related engagement through various psychological processes, including self-regulation deficits, reward sensitivity, and limitations in cognitive control (Griffiths, 2005; Kuss and Griffiths, 2017). The literature further discusses how both intrinsic motives, such as curiosity or learning interest, and extrinsic reinforcers, including social approval or immediate feedback, may shape individuals' general orientations toward digital technologies (Brand et al., 2014; Caplan, 2002). In the context of AI, continuous accessibility and adaptive personalization have been described as factors that may intensify user engagement cycles (Richet, 2025). Similarly, human-like conversational features and instant responsiveness have been linked to increased perceived effectiveness and higher usage frequency (Troshani et al., 2021). Importantly, these mechanisms are discussed within existing theoretical frameworks as potential contributors to habitual or uncontrolled patterns of GAI tool use, particularly when self-regulatory capacities are limited, rather than as variables directly examined in the present study.

Therefore, the present study aims to fill the gap identified in the literature by developing the Generative Artificial Intelligence Addiction Scale (GAI-AS). This scale is designed to measure dimensions such as loss of control, prioritization, deprivation, mood regulation, and functional impairment in individuals' interactions with GAI systems. The scale items were developed by considering both behavioral addiction components and specific psychological processes. Content validity was ensured through expert opinions, after which the structure was tested using exploratory and confirmatory factor analyses (Brand et al., 2019). The scale's convergent and discriminant validity, internal consistency reliability, and measurement invariance were evaluated. As a valid tool, the GAI-AS is expected to provide a solid foundation for studies examining the relationships between GAI use and variables such as loneliness, stress, FoMO, anthropomorphism tendency, and usage duration. Furthermore, it is anticipated to serve as a valuable tool for risk screening, awareness programs, and preventive interventions in the fields of education and counseling (Montag and Elhai, 2025; Richet, 2025).

Growing reliance on GAI tools has raised concerns about emerging patterns of excessive, compulsive, or dysregulated use among undergraduate students. Understanding the extent to which pre-service and in-service learners exhibit GAI-related addictive tendencies, as well as the demographic and usage factors associated with these tendencies, is crucial for informing the responsible integration of GAI in higher education. Hence, in the current study, the following research questions were addressed, and the corresponding hypotheses were tested.

1. Is the GAI-AS a valid and reliable instrument for assessing GAI-related addictive proneness among undergraduate students?
2. Do students' GAI addiction scores differ based on;

a. Gender,
b. Grade level,
c. The type of GAI version used (free vs. paid),
d. Frequency of GAI use?

Corresponding hypotheses:

H1: The students' GAI addiction scores differ by gender, grade level, GAI version, and frequency of GAI use.

3. What are the relationships among the four dimensions of the GAI-AS?

H2: Significant positive correlations exist among the four dimensions of the GAI-AS.

## 2 Methodology

The study was conducted in two sequential phases, consisting of scale development and large-scale implementation. In the first phase, the GAI-AS was developed through a systematic scale-development process (DeVellis, 2017). In the second phase, following confirmation of its psychometric adequacy, the GAI-AS was administered within a non-experimental descriptive study to assess students' levels of GAI-related addictive proneness.

### 2.1 Participants

The study involved three independent groups of undergraduate students who participated during the scale development (EFA and CFA) and the large-scale implementation phases. The scale was administered online through Google Forms, which was shared via direct links and QR codes. The instrument included a screening item on GAI use, and sixteen students who indicated that they did not use GAI tools were excluded from the analysis. The final sample consisted of students who met this criterion, and participation was anonymous, voluntary, and unpaid in all phases. Descriptive information about the participants in each phase is presented in Table 1.

**Table 1:** Demographics of the participants for three different processes.

Variables		Development phases				Large-scale implementation phases	
		EFA		CFA			
		*N*	%	*N*	%	*N*	%
	Total	429	100%	490	100%	803	100%
Gender	Female	263	61.3%	280	57.1%	494	61.5%
	Male	166	38.7%	210	42.9%	309	38.5%
Grade level	Freshman (I)	96	25.2%	121	24.7%	205	25.5%
	Sophomore (II)	106	27.7%	129	26.3%	264	32.9%
	Junior (III)	92	24.1%	128	26.1%	138	17.2%
	Senior (IV)	88	23.0%	112	22.9%	196	24.4%
Version	Only free	368	85.8%	402	82.0%	698	86.9%
	Free and paid	61	14.2%	88	18.0%	105	13.1%
GAI usage frequency	Every day (I)	125	29.1%	112	22.9%	227	28.3%
	4–6 days a week (II)	118	27.5%	133	27.1%	207	25.8%
	1–3 days a week (III)	112	26.1%	144	29.4%	277	34.5%
	Less than once a week (IV)	74	17.2%	101	20.6%	92	11.5%

The GAI-AS was administered to 429 students for EFA and 490 students for CFA, and later applied to an additional group of 802 students during the large-scale implementation phase.

### 2.2 GAI-AS development phase

**Figure 1:**
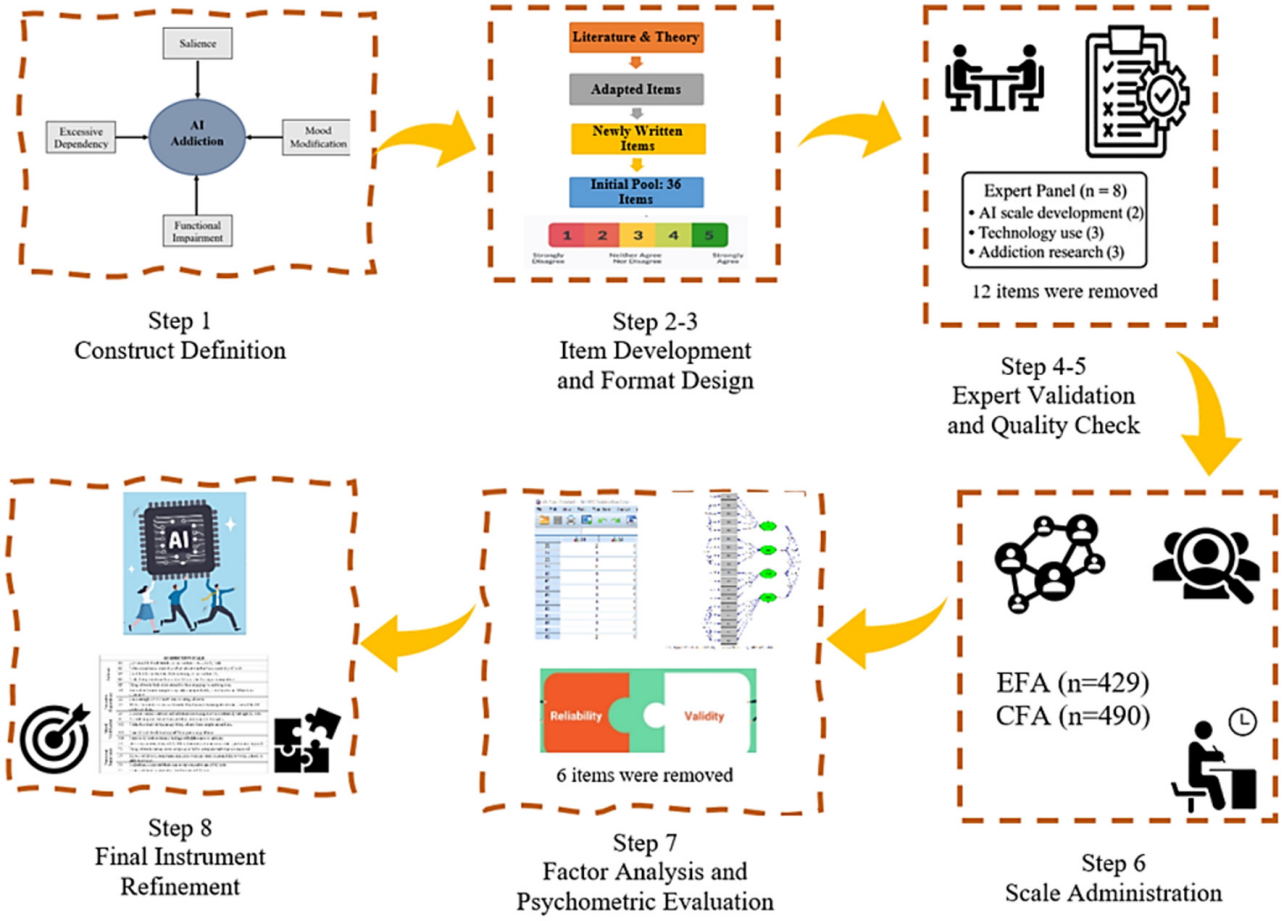
Development process of the GAI-AS based on DeVellis (2017).

The development of the GAI-AS followed the eight-step scale construction framework proposed by DeVellis (2017), which offers a systematic process for defining a construct, generating and refining items, and validating the resulting instrument (Figure 1). In the first step, the construct targeted by the scale was clearly defined to ensure a solid theoretical foundation for item development. The GAI-AS was designed to assess GAI addiction, described as a persistent and excessive use of GAI tools that interferes with cognitive, emotional, and functional aspects of daily life (Griffiths, 2005). Guided by contemporary perspectives on behavioral and technology-related addictions, the construct was organized into four dimensions reflecting key manifestations of problematic GAI use. Salience captures the extent to which GAI becomes central in an individual's daily thinking and routines. Excessive Dependency reflects a strong reliance on GAI for cognitive or instrumental tasks at the expense of one's own effort. Mood Modification refers to the use of GAI tools to cope with stress or regulate emotions. Functional Impairment encompasses the loss of control and the negative academic, social, or personal consequences resulting from excessive GAI engagement. Together, these dimensions provide a coherent framework for understanding GAI-related addictive behaviors.

In the second step, the initial item pool was created by reviewing well-known technology and behavioral addiction scales in the literature, including frameworks and items from studies on internet, smartphone, and gaming addictions (Griffiths, 1995; Kuss et al., 2013; Kwon et al., 2013). Recent research on digital tool overuse also informed the development of the items (Hu et al., 2023; Maral et al., 2026; Yu et al., 2024). Based on these sources, items reflecting cognitive involvement, emotional reliance, excessive use, and functional impairment were drafted, and the researchers created several additional items to ensure comprehensive coverage of GAI-related addictive behaviors. Following the development of the 36 preliminary items, the response format of the scale was determined. In this context, a total of 36 preliminary items were generated. After generating this pool, in the third step, the response format of the scale was determined. A five-point Likert-type structure was adopted, which is widely used in psychological and behavioral measurement (Judd et al., 1991). Each item was rated on a scale ranging from 1 (Strongly disagree) to 5 (Strongly agree).

In the fourth step, the preliminary set of 36 items was evaluated through expert review to ensure content clarity and conceptual alignment with the four-factor structure. The items were evaluated by a panel of eight experts, including two with experience in GAI-related scale development, three specializing in technology use, and three with expertise in addiction research. Experts reviewed the items for clarity, relevance, and conceptual alignment with the four-factor structure. Based on expert feedback, 12 items were removed due to redundancy or limited conceptual clarity. For example, one of the items retained in the pool expressed a strong emotional dependence on GAI tools: “*Life would feel unbearable without GAI tools in my daily routine*.” During the review, a second item with almost the same meaning, “*I cannot imagine living my life without constant access to GAI tools*,” was deemed unnecessarily repetitive. In the fifth step, the potential inclusion of validity-check items was considered to ensure the quality and consistency of participant responses. Such items help identify inattentive or patterned responding during administration. For this purpose, a simple verification item was included, asking participants to indicate their level of agreement with the statement “*Three plus five is eight*.” As an additional precaution, participant responses were also screened for missing data patterns, straight-lining, inconsistent responses, and unusually rapid completion times to strengthen the overall response-quality control process.

In the sixth step, the 24-item version of the scale was administered to the development sample. In this step, the GAI-AS was administered to a development sample to evaluate the structure derived from the earlier phases. There is no single consensus in the literature regarding the optimal number of participants required for scale validation; yet, many scholars emphasize the need for a sample that is sufficiently large in proportion to the number of items to ensure stable factor structures. Some methodological sources recommend using a sample several times larger than the total item count, while others note that samples approaching or exceeding 300 participants provide a solid foundation for factor analytic procedures (Comrey and Lee, 2013; Hair et al., 2010). Consistent with sample size recommendations for factor analysis, the administration involved two independent groups: 429 undergraduate students participated in the EFA phase and 490 undergraduate students in the CFA phase, both drawn from the larger pool of eligible respondents who confirmed active GAI use.

In the seventh step, item performance was examined through exploratory factor analysis, and an EFA was conducted using the SPSS program (IBM SPSS version 27.0). Following factor-analytic criteria (Hair et al., 2010), six items were removed. The four-dimensional structure and this configuration were subsequently tested through confirmatory factor analysis performed in LISREL. Model fit was evaluated using multiple goodness-of-fit indicators. These included absolute fit indices, namely the chi-square/df ratio, RMSEA, SRMR, GFI, and AGFI, and incremental fit indices, namely CFI and NFI (Brown, 2006; Hu and Bentler, 1999). Convergent and discriminant validity were examined through Composite Reliability and Average Variance Extracted values (Fornell and Larcker, 1981; Hair et al., 2010). Internal consistency was assessed using Cronbach's alpha, McDonald's Omega coefficients (Nunnally, 1978) and corrected item–total correlations (Aday and Cornelius, 2006). Additionally, Harman's single-factor test was employed to assess the potential for common method bias (Podsakoff et al., 2003). These analyses resulted in a refined instrument consisting of 18 items grouped under four factors, demonstrating strong validity and reliability for subsequent. The factor structure identified through EFA was then evaluated with CFA to confirm its adequacy using the LISREL program. Standard fit indices commonly recommended in the literature were examined to evaluate the adequacy of the CFA model (Brown, 2006; Hair et al., 2010; Hu and Bentler, 1999). Additional validity and reliability analyses were conducted, including the Composite Reliability (CR) and Average Variance Extracted (AVE) tests (Fornell and Larcker, 1981; Hair et al., 2010) for convergent and discriminant validity, Cronbach's alpha, McDonald's Omega and item-total correlations for internal consistency (Aday and Cornelius, 2006; Nunnally, 1978), and a Harman single-factor check to rule out common-method bias (Podsakoff et al., 2003). In the final step, the scale was optimized by retaining the most theoretically coherent and statistically robust items, resulting in an 18-item instrument representing four distinct factors.

### 2.3 Large-scale implementation phase of the GAI-AS

The final 18-item version of the GAI-AS was administered to a larger group of undergraduate students (*N* = 803) using an online Google Form, which was shared through direct links and QR codes. All analyses were conducted using the SPSS program. Reliability analyses demonstrated strong internal consistency for each factor (α values ranging from.81 to.89) and for the overall scale (α = 0.90). Prior to inferential analyses, the distributional properties of the data were examined through skewness–kurtosis values (±1 threshold) and the Kolmogorov-Smirnov test, indicating acceptable normality. Descriptive statistics, including mean scores for each dimension, were also considered to support the interpretation of the results. Group differences were tested using independent-samples *t*-tests and one-way ANOVA, followed by Tukey *post-hoc* comparisons when necessary. Effect size statistics were calculated to assess the practical significance of observed differences.

## 3 Results

### 3.1 Content validity

The preliminary 36-item pool [M1–M36] of the GAI-AS was reviewed by a panel of experts, who evaluated each item in terms of clarity, conceptual relevance, and alignment with the four proposed dimensions of GAI addiction. Based on their qualitative feedback, items deemed ambiguous, conceptually weak, or redundant were removed or rephrased. This expert review process, conducted in line with established guidelines for content validation (Lynn, 1986; Polit and Beck, 2006), resulted in the elimination of 12 items and refinement of the remaining statements. The final pool consisted of 24 items judged to adequately and coherently represent the cognitive, emotional, and behavioral components of GAI addiction.

### 3.2 Construct validity

The construct validity of the GAI-AS was examined through EFA and CFA.

#### 3.2.1 Exploratory factor analysis

**Figure 2:**
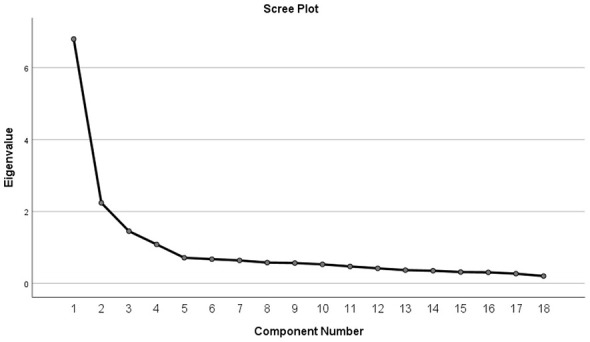
Scree plot of GAI-AS.

Descriptive statistics demonstrated adequate item variability, with means ranging from 1.59 to 3.51 and standard deviations between 0.93 and 1.28. The skewness and kurtosis coefficients were within acceptable limits for factor analysis, indicating no problematic deviations from distributional assumptions, which is consistent with the recommended criteria (Kline, 2005). The suitability of the data for factor analysis was further supported by a high KMO value (0.904) and a significant Bartlett's Test of Sphericity [χ^2^ (153) = 3,627.52, *p* < 0.001], meeting established guidelines for sampling adequacy and factorability (Tabachnick and Fidell, 1996). Accordingly, EFA was performed using principal axis factoring with direct oblimin rotation, based on the expectation that the dimensions would be interrelated (Costello and Osborne, 2005). A minimum loading criterion of 0.50 and a cross-loading threshold of <0.10 (Hair et al., 2010) guided item retention. Based on the item retention criteria, six items were removed from the initial pool, yielding an 18-item scale. As shown in Figure 2, the scree plot supported the factor retention decision, and the remaining items demonstrated strong and interpretable factor loadings.

Examination of eigenvalues greater than 1 then supported a four-factor structure, which together accounted for 64.32% of the total variance, indicating a substantial representation of the underlying construct. The items loaded clearly on four coherent dimensions, with factor loadings ranging from 0.569 to 0.833 (Table 2).

**Table 2:** The item factor loads, and the explained total variance of the GAI-AS.

Items	Salience	Excessive dependency	Mood modification	Functional impairment	Total variance
S1	0.720				37.70%
S2	0.569				
S3	0.790				
S4	0.816				
S5	0.794				
E1		0.762			12.44%
E2		0.712			
E3		0.646			
E4		0.735			
M1			0.600		8.07%
M2			0.729		
M3			0.738		
M4			0.785		
F1				0.786	6.02%
F2				0.785	
F3				0.833	
F4				0.824	
F5				0.737	
Total					64.31%

The final pattern matrix showed a well-defined distribution of items across factors, providing robust empirical support for the multidimensional structure of the GAI-AS. CFA was subsequently performed to evaluate the adequacy of the four-factor, 18-item structure of the GAI-AS derived from the exploratory phase (Appendix 1).

#### 3.2.2 Confirmatory factor analysis

**Figure 3:**
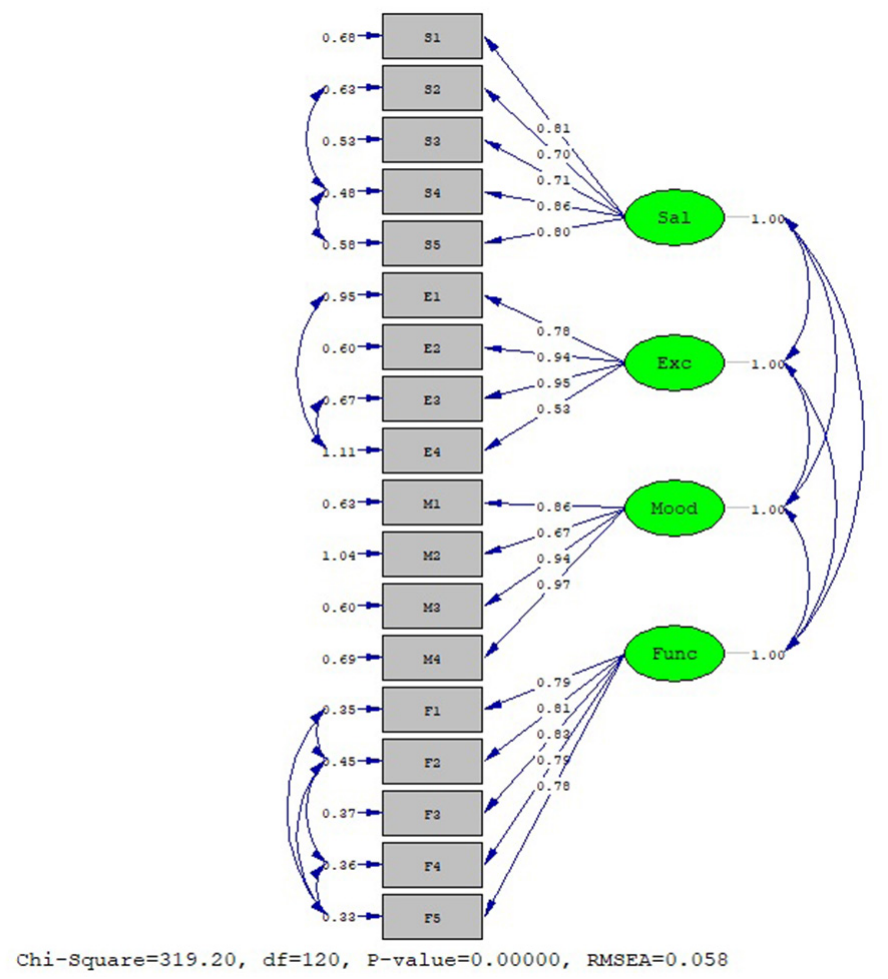
Standardized factor loadings from the CFA model of the GAI-AS.

The analysis yielded robust item-factor relationships, with standardized loadings ranging from 0.53 to 0.97. The model demonstrated acceptable fit to the data, with $\chi_{\left(113\right)}^{2}$ = 319.20, χ^2^/df = 2.66, RMSEA = 0.058, SRMR = 0.051, and RMR = 0.063. Incremental fit indices were high (CFI = 0.98, IFI = 0.98, NFI = 0.97), while absolute fit indices (GFI = 0.93, AGFI = 0.90) also fell within acceptable ranges, together supporting the adequacy of the proposed model (Brown, 2006; Hair et al., 2010; Hu and Bentler, 1999; Kline, 2005). All factor loadings were statistically significant (*p* < 0.001), and their standardized values exceeded the commonly accepted threshold of 0.50, indicating that each item made a meaningful contribution to its corresponding latent construct (Hair et al., 2010) (Figure 3). Modification indices were examined following the initial CFA to identify localized sources of misfit. Correlated error terms were introduced only for theoretically justifiable item pairs within the same factor, as presented in the CFA model, and no cross-factor error covariances were allowed. These adjustments improved the χ^2^ value and overall model fit indices.

#### 3.2.3 Convergent and discriminant validity

Convergent validity was assessed using composite reliability (CR) and average variance extracted (AVE) in accordance with the guidelines proposed by Hair et al. (2010) and Fornell and Larcker (1981). Across the four dimensions of the scale, CR values ranged from 0.884 to 0.923, while AVE values varied between 0.605 and 0.753, exceeding the commonly accepted thresholds of 0.70 for CR and 0.50 for AVE. Specifically, the salience dimension demonstrated a CR of 0.884 and an AVE of 0.605, excessive dependency yielded a CR of 0.885 with an AVE of 0.668, mood modification exhibited the highest levels of internal consistency with a CR of 0.923 and an AVE of 0.753, and functional impairment showed a CR of 0.898 alongside an AVE of 0.640. These results confirm that the indicators consistently represent their respective constructs, providing strong evidence of convergent validity for the measurement model.

Discriminant validity was evaluated using the Fornell–Larcker criterion (Fornell and Larcker, 1981). According to this approach, each construct should explain more variance in its own indicators than it shares with other constructs (Table 3).

**Table 3:** Discriminant validity of the GAI-AS.

Dimensions	Salience	Excessive dependency	Mood modification	Functional impairment
Salience	**0.77**	0.75	0.68	0.66
Excessive dependency	0.75	**0.81**	0.70	0.45
Mood modification	0.68	0.70	**0.86**	0.53
Functional impairment	0.66	0.45	0.53	**0.80**

As shown in this table, the AVE values for all dimensions were greater than their corresponding inter-construct correlations, indicating that discriminant validity was satisfactorily established. Bold values on the diagonal represent the square roots of the AVE for each dimension.

### 3.3 Internal consistency

Internal consistency indicators for the GAI-AS are presented in Table 4, including corrected item-total correlations, Cronbach's alpha coefficients, and McDonald's omega values.

**Table 4:** Internal consistency of GAI-AS.

Dimensions	Corrected item-total correlation	Cronbach's alpha	McDonald's omega
Salience	0.405–0.600	0.82	0.88
Excessive dependency	0.325–0.560	0.81	0.83
Mood modification	0.341–0.646	0.83	0.88
Functional impairment	0.485–0.654	0.88	0.89
GAI-AS total	0.325–0.654	0.90	0.91

Cronbach's alpha and McDonald's omega coefficients indicated consistently strong reliability across all four subscales, with values ranging from 0.81 to 0.88 for alpha and 0.83 to 0.91 for omega, demonstrating that each dimension of the GAI-AS is represented by a highly coherent set of items (Nunnally, 1978). The total scale also demonstrated excellent internal consistency, with an alpha coefficient of 0.90 and an omega coefficient of 0.91. Corrected item-total correlations fell within theoretically appropriate ranges for all dimensions (0.325–0.654), demonstrating that each item contributed meaningfully to the construct it was designed to assess (Aday and Cornelius, 2006). These indicators demonstrate that the scale exhibits strong internal consistency and functions as a psychometrically sound instrument.

### 3.4 Common method bias analysis

To evaluate potential common method bias, a confirmatory factor analysis was conducted using Harman's single-factor procedure, in which all GAI-AS items were constrained to load onto a single latent construct. Exploratory Factor Analysis (EFA) and Confirmatory Factor Analysis (CFA) were conducted using Linear Structural Relations (LISREL). Model fit was evaluated using the Root Mean Square Error of Approximation (RMSEA), Standardized Root Mean Square Residual (SRMR), Goodness-of-Fit Index (GFI), Adjusted Goodness-of-Fit Index (AGFI), Comparative Fit Index (CFI), Normed Fit Index (NFI), Incremental Fit Index (IFI), Parsimony Goodness-of-Fit Index (PGFI), and Root Mean Square Residual (RMR). Prior to factor analyses, the Kaiser–Meyer–Olkin (KMO) Measure of Sampling Adequacy was examined. Analysis of Variance (ANOVA) was used to compare group means. The resulting one-factor model showed a clearly inadequate fit [$\chi_{\left(135\right)}^{2}$ = 2,053.57, RMSEA = 0.170, GFI = 0.68, AGFI = 0.60, SRMR = 0.098, RMR = 0.12, PGFI = 0.54], indicating that the covariance among items could not be explained by a single underlying factor. Consistent with these findings, the first factor extracted in the exploratory factor analysis accounted for only 37.70% of the total variance, which is below the 50% threshold suggested by Podsakoff et al. (2003). These results provide strong evidence that common method bias did not meaningfully affect the GAI-AS data.

### 3.5 Implementation of the GAI-AS

**Figure 4:**
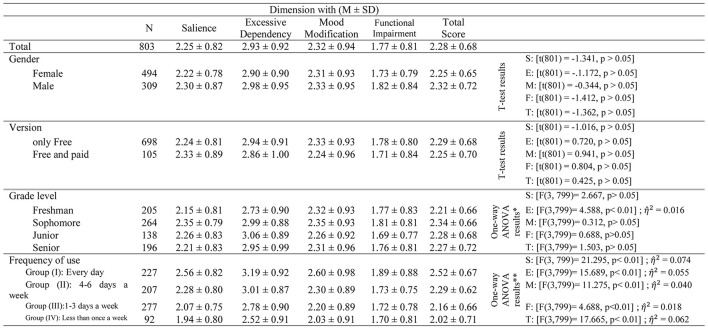
Summary of descriptive statistics and statistical results for the implementation stage. *Post-hoc comparisons: Tukey HSD indicated that in the Excessive Dependency on AI Tools dimension, Freshman students scored significantly lower than Sophomore, Junior, and Senior students. **Post-hoc comparisons: Tukey HSD showed that for all four dimensions of the AI-AS and for the total scale score, Group I scored significantly higher than Groups II, III, and IV; Group II scored higher than Groups III and IV; and Group III scored higher than Group IV. *M*, mean; SD, standard deviation; *N*, number of participants; S, salience; E, excessive dependency; M, mood modification; F, functional impairment.

This section reports the findings obtained from the statistical analyses conducted on the GAI-AS. The variation in participants' total and subscale scores across grade level, GAI version used, and frequency of use is presented in Figure 4. The GAI-AS scores did not differ significantly by gender across any of the four dimensions or the total scale score. The mean scores of female students were similar to those of male students in Salience, Excessive Dependency, Mood Modification, Functional Impairment, and overall GAI-AS scores, and all independent-samples *t*-tests were non-significant (*p* > 0.05). These results indicate that gender did not influence participants' GAI addiction scores. In addition, comparisons between students using only the free version of the GAI tool and those using both free and paid versions showed no statistically significant differences in any of the four dimensions of the GAI-AS or in the total addiction score (*p* > 0.05). These findings suggest that the type of GAI version accessed by participants did not influence their levels of GAI-related addictive tendencies.

Regarding grade level, statistically significant differences were found in the Excessive Dependency dimension, whereas no meaningful variation was found in Salience, Mood Modification, Functional Impairment, or the total score. Freshman students (M = 2.73, SD = 0.90) reported significantly lower excessive dependency compared with Sophomore (M = 2.99, SD = 0.88), Junior (M = 3.06, SD = 0.89), and Senior students (M = 2.95, SD = 0.99), *F* (3,799) = 4.588, *p* < 0.01; η^2^ = 0.016. The small effect size indicates that although Freshman students showed lower excessive reliance on GAI tools than upper-grade students, the difference was modest. The non-significant results for the remaining dimensions and the total score further indicate that grade level did not systematically account for variance in broader GAI addiction indicators.

Moreover, with respect to frequency of GAI use, statistically significant differences were observed across all four dimensions of the GAI-AS as well as the total scale score. Participants who reported using GAI tools every day demonstrated the highest levels of Salience, Excessive Dependency, Mood Modification, Functional Impairment, and the overall GAI addiction score. ANOVA results confirmed significant group differences for Salience, *F* (3,799) = 21.295, *p* < 0.01, η^2^ = 0.074; Excessive Dependency, *F* (3,799) = 15.689, *p* < 0.01, η^2^ = 0.055; Mood Modification, *F* (3,799) = 11.275, *p* < 0.01, η^2^ = 0.040; Functional Impairment, *F* (3,799) = 4.688, *p* < 0.01, η^2^ = 0.018; and the total score, *F* (3,799) = 17.665, *p* < 0.01, η^2^ = 0.062. *Post-hoc* analyses showed a consistent stepwise pattern in which daily users scored significantly higher than all other groups, users engaging 4–6 days per week scored higher than those using GAI 1–3 days per week and less than once a week, and users in the 1–3 days' group scored higher than those in the lowest-frequency group. Taken together, these results indicate that more frequent engagement with GAI tools is consistently associated with higher levels of GAI-related addictive proneness, with effect sizes ranging from small to moderate. Besides, a Pearson correlation analysis was conducted to examine the relationships among the four dimensions of the GAI-AS. The results showed that all sub-dimensions were positively and significantly correlated (*p* < 0.01). Salience demonstrated moderate positive associations with Excessive Dependency (*r* = 0.530), Mood Modification (*r* = 0.521), and Functional Impairment (*r* = 0.536). Excessive Dependency was moderately correlated with Mood Modification (*r* = 0.525) and weak-to-moderately correlated with Functional Impairment (*r* = 0.337). Additionally, a moderate positive correlation was found between Mood Modification and Functional Impairment (*r* = 0.519). Overall, participants who scored higher on one dimension of GAI addiction also tended to score higher on the other dimensions of the GAI-AS.

## 4 Discussion

In today's digitally driven world, understanding the evolving relationship between humans and GAI is essential for navigating this rapidly transforming landscape responsibly. GAI tools are now deeply embedded in individuals' daily routines, reshaping activities ranging from academic tasks to professional decision-making. Although these tools offer substantial cognitive ease, they simultaneously carry risks that may undermine emotional and mental health. This dual nature introduces a range of risks, including negative effects on both academic performance and physiological and psychological wellbeing (Montag and Ali, 2025; Naseer et al., 2025). Excessive or compulsive engagement with such tools may resemble behavioral addiction, highlighting the need for valid and reliable instruments intended to identify GAI addiction. Therefore, the need for measurement instruments that are easy to understand and can be easily completed by undergraduate students is necessary for measuring GAI addiction. Hence, the primary aim of the current study is to develop a valid and reliable scale to conceptualize and assess GAI addiction among undergraduate students. In line with this aim, the content validity of the GAI-AS was established through expert panel reviews; its construct validity was examined using EFA, CFA, and assessments of convergent and discriminant validity; and its internal consistency was calculated using Cronbach's alpha and McDonald's omega coefficients.

The initial item pool of 36 items was reduced to 24 items after the expert panel review process. The EFA results subsequently demonstrated a well-defined four-factor structure for the 18-item GAI-AS, with factor loadings ranging from 0.569 to 0.833 and a total explained variance of 64.32%. Moreover, the CFA results demonstrated very good or close-to-good fit indices, indicating an overall well-fitting model in which all items showed significant factor loadings that confirm their substantive contribution to representing the construct (Brown, 2006; Hair et al., 2010; Hu and Bentler, 1999; Kline, 2005). Given that prior GAI-related dependence scales have reported total variance values between 40 and 74%, the total variance explained by the 18-item GAI-AS is consistent with the acceptable range reported in the literature (Akinwale et al., 2025; Castro et al., 2025; Chen et al., 2025; Morales-García et al., 2024; Zhang et al., 2025a). In the GAI-AS, all items exceeded the commonly accepted factor-loading threshold of 0.50 (Hair et al., 2010). In line with Whittaker and Worthington's (2016) assertion that higher loadings refer to stronger relationships with the latent construct, these findings provide robust evidence that each item makes a meaningful contribution to the underlying factor structure.

Moreover, convergent and discriminant validity were satisfactorily established, providing strong evidence of the scale's validity (Hair et al., 2010; Fornell and Larcker, 1981). Similarly, Harman's single-factor test indicated that common method bias did not meaningfully influence the GAI-AS data (Podsakoff et al., 2003). Lastly, reliability analyses indicated strong internal consistency for the GAI-AS, evidenced by a Cronbach's alpha of 0.90 and a McDonald's omega coefficient of 0.91 (Aday and Cornelius, 2006; Nunnally, 1978). Overall, these findings demonstrate that the GAI-AS is a valid and reliable instrument with four factors of 18 items for assessing GAI addiction levels among undergraduate students.

The second aim of our study is to reveal the effect of several demographic characteristics on GAI-AS. Based on the GAI-AS results for age and version of GAI tools (free vs. paid), no statistically significant differences were found among undergraduate students. Similarly, the lack of significant gender differences reported by Robayo-Pinzon et al. (2025) supports our findings, although some studies have noted higher levels of GAI dependence among men (Chen et al., 2025; Estrada-Araoz et al., 2025). Future research should further investigate this phenomenon to clarify the complex ways in which user perceptions interact with emerging forms of technological integration. Additionally, the lack of differences in dependency between users of free and paid versions of GAI tools can be attributed to the fact that dependency is more closely linked to the frequency of use than to the level of access. This may be explained by the fact that the free version already includes core features (e.g., instant feedback, reducing cognitive load, generating quick solutions, personalized responses) capable of eliciting the same reinforcement and habitual use patterns as the paid version.

The study also identified significant differences based on the frequency of use, with undergraduate students who reported higher GAI usage exhibiting higher overall dependence levels and higher scores across all GAI-AS dimensions than those who used GAI less frequently. This result aligns with Robayo-Pinzon et al. (2025), who reported higher dependence levels among 2–3-h GAI users compared to those using the tools for 1–2 h or less. The increase in addiction tendencies with higher GAI usage frequency may be explained by the fact that GAI systems provide immediate feedback (Montag et al., 2021), offer personalized responses that strengthen feelings of personal connection, emotional comfort, and perceived support (Skjuve et al., 2023), and give users a sense of obtaining solutions quickly and with minimal effort (Schei et al., 2024). Similarly, several researchers noted that repeated engagement with digital platforms reinforces reward mechanisms, which may lay the groundwork for behavioral addiction (Griffiths, 2005; Montag and Reuter, 2015).

Furthermore, by grade level, only the Excessive Dependency dimension showed significant differences, with freshman students scoring significantly lower than sophomore, junior, and senior students. This may be explained by the types of assignments students undertake, as first-year students typically complete tasks that differ in structure and cognitive demands from those required in later academic years. In this sense, their higher dependence on GAI tools may reflect a more instrumental reliance on AI for managing demanding academic workloads rather than a simple lack of autonomy. This interpretation is consistent with da Silva's (2024) view that assignments requiring personal opinions and critical evaluation may reduce students' reliance on generic AI-generated responses. It also aligns with Özbay's (2026) finding that older university students report lower levels of AIlessphobia and greater academic autonomy. Taken together, these findings suggest that frequent GAI use among upper-class students does not necessarily indicate stronger psychological vulnerability. Rather, as students gain academic experience, they may become more capable of using AI strategically and selectively, supported by stronger self-regulation, ethical awareness, and academic agency (Liu et al., 2025; Özbay, 2026).

The positive and significant correlations observed among the four dimensions of the GAI-AS suggest that salience, excessive dependency, mood modification, and functional impairment function as interrelated components of a multidimensional behavioral construct. These findings can be further understood by situating GAI addiction within broader psychological risk factors. Recent work suggests that problematic AI use may be closely related to AIlessphobia (Gezgin and Türk Kurtça, 2025; Özbay, 2026). In this respect, the Excessive Dependency and Mood Modification dimensions of the GAI-AS may reflect not only frequent use, but also an underlying need for emotional reassurance. Students with lower academic self-efficacy may turn to GAI tools to reduce academic anxiety, yet this reliance can gradually weaken confidence in their own cognitive abilities. Thus, the use of GAI for emotional regulation may become an early pathway to functional impairment, particularly when these tools are used as a substitute for students' own learning efforts rather than as supportive aids. This pattern of moderate associations is also consistent with Griffiths' (2005) components model of addiction, which views addictive behaviors as a set of dynamically connected processes. In particular, the links between mood modification, salience, and functional impairment suggest that emotional regulation motives may play a central role in sustaining intensive GAI engagement and its negative consequences. Overall, these theoretically coherent interrelations provide further support for the internal construct validity of the GAI-AS.

Despite the strong psychometric evidence supporting the GAI-AS, it remains important to critically consider whether problematic GAI use should be conceptualized as a distinct behavioral disorder. As Ciudad et al. (2025) cautions, dysregulated engagement with GAI may not yet meet conventional diagnostic criteria for clinical addiction. Rather, such use may represent a compensatory coping strategy, particularly among students who experience academic insecurity or low self-efficacy. For example, students with limited confidence in their academic abilities may become overly dependent on GAI systems as a way to reduce feelings of inadequacy, avoid academic discomfort, or manage performance-related anxiety (Özbay, 2026). Accordingly, future research should continue to examine the conceptual and clinical boundaries of GAI dependence, clarifying whether it develops into an independent form of behavioral pathology or remains better understood as a maladaptive, technology-mediated coping mechanism.

Although the present study offers important insights, future research should incorporate additional contextual and psychosocial variables (e.g., economic conditions, urban-rural context, sleep quality, the social substitution effect, mental health risks, wellbeing, etc.). Prior studies indicate that these factors substantially influence technology-related behavioral outcomes, suggesting that their omission may limit the explanatory scope of the current findings. For instance, socioeconomic and geographical disparities influence susceptibility to technology dependence (Lee and McKenzie, 2015), while elevated screen use is consistently associated with reductions in sleep quality (Santos et al., 2023). Likewise, overreliance on technology-mediated interactions may erode real-world support networks (Crawford et al., 2024), and excessive AI use as an avoidance-based coping strategy may increase emotional vulnerability and may ultimately increase the risk of mental health issues (Chen et al., 2025; Hu et al., 2023; Zhang et al., 2025b). Nevertheless, GAI addiction is a new phenomenon and differs across cultures can be examined in future studies, with cross-cultural comparison studies applying both qualitative and quantitative research methods. Additionally, whether GAI addiction varies according to individual differences, personality traits, cognitive factors, social factors, and emotional and psychological factors can be investigated in depth through longitudinal research studies. Integrating these variables into future designs would enable the development of more comprehensive and methodologically rigorous models, capable of capturing the multidimensional nature of GAI addiction.

## 5 Limitations

The current study presents noteworthy outcomes, however, several limitations should be acknowledged when interpreting the results. First, our sample consisted of undergraduate students, who are generally more inclined to adopt new technologies and widespread use of GAI applications and is not representative of the entire population. Thus, the lack of demographic diversity within the working group may have limited the broader applicability of our findings. Second, the study's cross-sectional design presents an important limitation. Although this approach offers valuable theoretical insights, the data were collected at a single point in time, which restricts the ability to draw causal inferences among the variables examined. In particular, it remains unclear whether frequent GAI use leads to higher addiction tendencies or whether individuals with stronger addiction tendencies are more likely to use GAI applications frequently. Future research employing longitudinal or experimental designs would therefore provide a more robust understanding of the temporal and causal dynamics underlying GAI addiction with more heterogeneous samples drawn from diverse cultural, educational, and demographic backgrounds. Another limitation of this study is that criterion-related validity was not tested. Although several psychometric analyses were conducted, the GAI-AS was not compared with existing scales of problematic technology use, internet addiction, smartphone addiction, or related AI-use constructs. Future studies should examine these relationships to provide stronger evidence for the scale's validity. They should also test concurrent and predictive validity and explore whether GAI-AS scores remain stable over time. Given that the participants were undergraduate students, future studies should also examine how GAI addiction is associated with educational outcomes, such as academic self-efficacy, academic performance, learning satisfaction, critical thinking, and self-regulated learning. This would provide a clearer understanding of the educational implications of problematic GAI use.

Third, although this study focused on the overall conceptual structure of GAI addiction, it did not consider subtype-specific features (e.g., educational, social-interactive, leisure-oriented, productivity-focused, or creative-generative GAI applications). Hence, future studies should investigate how different GAI use contexts may produce distinct addiction profiles. Lastly, the GAI-AS relies on self-report data, which may be subject to social desirability, recall errors, and limited self-awareness, potentially affecting the precision of the responses. To address these concerns, future research could incorporate multi-method approaches- such as behavioral usage metrics, observational data, or informant reports- to triangulate findings and strengthen the validity of GAI addiction assessments.

## 6 Conclusion

To sum up, the current study developed the GAI-AS as a valid and reliable measurement instrument for undergraduate students, designed to assess GAI addiction and demonstrating strong psychometric properties. The GAI-AS consists of 18 five-point Likert items and includes four dimensions: salience, Mood Modification, Excessive Dependency, and Functional Impairment. In particular, its concise format facilitates easy incorporation and offers clear advantages due to its ease of administration and scoring.

The results indicate that all dimensions of GAI-AS differ significantly according to students' frequency of use. In contrast, no meaningful differences were observed with respect to age or GAI version (paid vs. free) among undergraduate students. Additionally, freshman students scored significantly lower on the Excessive Dependency dimension compared to sophomore, junior and senior students. Overall, the findings position the GAI-AS as a comprehensive measure of GAI addiction among undergraduates, indicating that future interventions and institutional policies should encourage longitudinal and predictive research to better understand how GAI-engagement patterns evolve across students' academic progression.

## Data Availability

The raw data supporting the conclusions of this article will be made available by the authors, without undue reservation.

## Appendix

**Table A1:** Generative artifical intelligence addiction scale.

Salience	S1	Life would feel unbearable for me without access to AI tools.
	S2	I often experience internal conflicts about whether I am overusing AI tools.
	S3	I feel that the internet has little meaning for me without AI.
	S4	Tasks that prevent me from using AI tools feel boring or unappealing.
	S5	Using AI tools feels more attractive than engaging in anything else.
Excessive dependency	E1	Even when I could complete my tasks independently, I tend to rely on AI tools for assistance.
	E2	I increasingly devote more time to using AI tools.
	E3	When I am unable to access AI tools, I find myself thinking about what I would do if I could use them.
	E4	I conduct online searches and information-seeking activities primarily through AI tools.
Mood modification	M1	AI tools help me escape from problems and negative thoughts.
	M2	I hide the extent or frequency of my AI use from people around me.
	M3	I use AI tools to distract myself from personal problems.
	M4	I turn to AI tools to reduce feelings of helplessness or anxiety.
Functional impairment	F1	Due to my increased use of AI, I have lost interest in activities that I previously enjoyed.
	F2	Using AI tools causes me to postpone or fail to complete tasks that are required of me.
	F3	My use of AI tools sometimes interferes with my other responsibilities (work, school, or daily activities).
	F4	I experience sleep problems due to my excessive use of AI tools.
	F5	I find it difficult to control or limit my use of AI tools.
